# CNS macrophages differentially rely on an intronic *Csf1r* enhancer for their development

**DOI:** 10.1242/dev.194449

**Published:** 2020-12-16

**Authors:** David A. D. Munro, Barry M. Bradford, Samanta A. Mariani, David W. Hampton, Chris S. Vink, Siddharthan Chandran, David A. Hume, Clare Pridans, Josef Priller

**Affiliations:** 1 UK Dementia Research Institute at The University of Edinburgh, Chancellor's Building, Edinburgh EH16 4SB, UK; 2 The Roslin Institute & Royal (Dick) School of Veterinary Sciences, The University of Edinburgh, Easter Bush, Midlothian EH25 9RG, UK; 3 Centre for Inflammation Research, Queen's Medical Research Institute, The University of Edinburgh, Edinburgh EH16 4TJ, UK; 4 Euan MacDonald Centre for MND Research, The University of Edinburgh, Edinburgh EH16 4SB, UK; 5 Centre for Clinical Brain Sciences, The University of Edinburgh, Edinburgh EH16 4SB, UK; 6 Anne Rowling Regenerative Neurology Clinic, The University of Edinburgh, Edinburgh EH16 4SB, UK; 7 Mater Research Institute, University of Queensland, Translational Research Institute, Woolloongabba Q4102, Australia; 8 The University of Edinburgh Centre for Inflammation Research, The Queen's Medical Research Institute, Edinburgh BioQuarter, 47 Little France Crescent, Edinburgh EH16 4TJ, UK; 9 Simons Initiative for the Developing Brain, Centre for Discovery Brain Sciences, The University of Edinburgh, Hugh Robson Building, George Square, Edinburgh EH8 9XD, UK; 10 Department of Neuropsychiatry and Laboratory of Molecular Psychiatry, Charité – Universitätsmedizin Berlin, 10117 Berlin, Germany

**Keywords:** Phagocytes, Myeloid cells, Kolmer cells, Cerebrospinal fluid, Cerebral ventricles, CNS-associated macrophages

## Abstract

The central nervous system hosts parenchymal macrophages, known as microglia, and non-parenchymal macrophages, collectively termed border-associated macrophages (BAMs). Microglia, but not BAMs, were reported to be absent in mice lacking a conserved *Csf1r* enhancer: the *fms*-intronic regulatory element (FIRE). However, it is unknown whether FIRE deficiency also impacts BAM arrival and/or maintenance*.* Here, we show that macrophages in the ventricular system of the brain, including Kolmer's epiplexus macrophages, are absent in *Csf1r*^ΔFIRE/ΔFIRE^ mice. Stromal choroid plexus BAMs are also considerably reduced. During normal development, we demonstrate that intracerebroventricular macrophages arrive from embryonic day 10.5, and can traverse ventricular walls in embryonic slice cultures. In *Csf1r*^ΔFIRE/ΔFIRE^ embryos, the arrival of both primitive microglia and intracerebroventricular macrophages was eliminated, whereas the arrival of cephalic mesenchyme and stromal choroid plexus BAMs was only partially restricted. Our results provide new insights into the development and regulation of different CNS macrophage populations.

## Abstract (precis)

**Summary:** Deletion of the *fms*-intronic regulatory element of *Csf1r* in mouse disrupts the engraftment and maintenance of central nervous system macrophages in a compartment-specific manner.

## INTRODUCTION

Macrophages are present throughout the central nervous system (CNS). The parenchymal macrophages of the CNS are known as microglia (RíoHortega, 1919), whereas the compartmentalised non-parenchymal macrophages are collectively termed border- or CNS-associated macrophages (BAMs or CAMs, respectively; Mrdjen et al., 2018; Kierdorf et al., 2019). BAMs reside within the tissue interfaces that separate CNS from periphery (such as the meninges, perivascular spaces and choroid plexus), where they seemingly function to safeguard the CNS by preventing infection and supporting barrier functions (Kierdorf et al., 2019). Recent fate-mapping and multi-omics studies have consistently demonstrated that microglia and BAMs are distinct cellular entities in terms of their development, capacity to self-renew, and their mRNA and protein expression profiles (Mrdjen et al., 2018; Jordão et al., 2019; Masuda et al., 2019; Van Hove et al., 2019; Utz et al., 2020).

In mice, evidence suggests that most microglia and certain BAM populations derive from primitive haematopoietic progenitors and/or erythromyeloid progenitors (EMPs), both of which emerge in the yolk sac (Ginhoux et al., 2010; Kierdorf et al., 2013; Gomez Perdiguero et al., 2015; Gomez Perdiguero and Geissmann, 2016; Utz et al., 2020). These cells enter the head and brain of the embryo as ‘pre-macrophages’ from approximately embryonic day (E) 9.5 (Ginhoux et al., 2010; Schulz et al., 2012; Mass et al., 2016) following the gradual establishment of the embryonic circulation (McGrath et al., 2003). Shortly thereafter, two transcriptionally distinct macrophage populations are found residing within the embryonic head (Utz et al., 2020). Most of those within the developing CNS parenchyma lack expression of the mannose receptor (*Mrc1*, the gene encoding CD206) and develop into TGF-β-dependent microglia, whereas primitive macrophages in the meningeal and cephalic (head) mesenchyme highly express *Mrc1* and become TGF-β-independent BAMs (Utz et al., 2020).

In adults, most BAMs share a core transcriptional signature, but there are also considerable compartment-specific transcriptomic differences in individual BAM subsets (Van Hove et al., 2019). BAMs within the stroma of the choroid plexus (ChP), for example, have distinct mRNA profiles compared with meningeal BAMs. Notably, a population of intracerebroventricular macrophages, which sit upon the ventricle-facing surface of the ChP (Kolmer's epiplexus macrophages; Kolmer, 1921), exhibit a transcriptional programme more consistent with microglia than BAMs. Furthermore, epiplexus macrophages share their ontogeny and self-renewal capacity with microglia, so were deemed as a non-parenchymal microglia subset (Van Hove et al., 2019). Intriguingly, macrophages floating within human cerebrospinal fluid also share transcriptional properties with microglia (Farhadian et al., 2018; Schafflick et al., 2020; Esaulova et al., 2020).

Microglia rely on signalling through the colony stimulating factor 1 receptor (CSF1R) for both their development and maintenance in adulthood (Erblich et al., 2011; Ginhoux et al., 2010). BAMs also rely on CSF1R-signalling for their maintenance in adulthood (Mrdjen et al., 2018; Van Hove et al., 2019), but it is unclear whether their development is CSF1R-dependent. Human *CSF1R* mutations have been associated with a range of neurological disorders, such as adult-onset leukoencephalopathy with axonal spheroids and pigmented glia (ALSP) (Rademakers et al., 2011; Guo et al., 2019; Oosterhof et al., 2019; Hume et al., 2020). Consequently, pharmacological and genetic tools have been developed to target CSF1R to better understand its roles in CNS diseases and macrophage biology (Han et al., 2017).

Recently, mutant mice were generated with genomic deletion of the *fms*-intronic regulatory element (FIRE) (Rojo et al., 2019), which is a highly conserved super-enhancer within the second intron of the *Csf1r* locus (Himes et al., 2001; Sasmono et al., 2003; Sauter et al., 2013). Microglia are absent from the brains of *Csf1r*^ΔFIRE/ΔFIRE^ mice, while some BAMs are retained (Rojo et al., 2019). Unlike other genetic models in which microglia are depleted, these mice have no overt brain development abnormalities and generally survive well into adulthood, making this a valuable tool to study these cells.

Here, we investigated the arrival dynamics of head and brain macrophages and explored the specificity of CNS macrophage loss in *Csf1r*^ΔFIRE/ΔFIRE^ mice. We show that microglia and intracerebroventricular macrophages are dependent on FIRE, whereas cephalic mesenchyme and stromal ChP BAMs are only partially reliant. We also demonstrate the arrival timing of intracerebroventricular macrophages, and present evidence suggesting that macrophages may enter ventricles by crossing the ventricular neuroepithelial lining during development.

## RESULTS AND DISCUSSION

### Intracerebroventricular and ChP macrophages differentially depend on FIRE during adulthood

In the initial organism-wide characterisation of adult *Csf1r*^ΔFIRE/ΔFIRE^ mice (Rojo et al., 2019), microglia were absent from brains, whereas BAMs were seemingly maintained. Some ChP BAMs were present (but not quantified), and adult intracerebroventricular macrophages, such as Kolmer's epiplexus cells, were not investigated. Thus, we decided to perform a detailed spatiotemporal analysis of the CNS myeloid cell compartment in *Csf1r*^ΔFIRE/ΔFIRE^ mice. First, we immunostained brain sections from adult *Csf1r*^ΔFIRE/ΔFIRE^ mice for a pan-macrophage marker, ionized calcium-binding adapter molecule 1 (IBA1; Köhler, 2007), to explore which CNS macrophage populations were impaired by FIRE deletion.

**Fig. 1.**
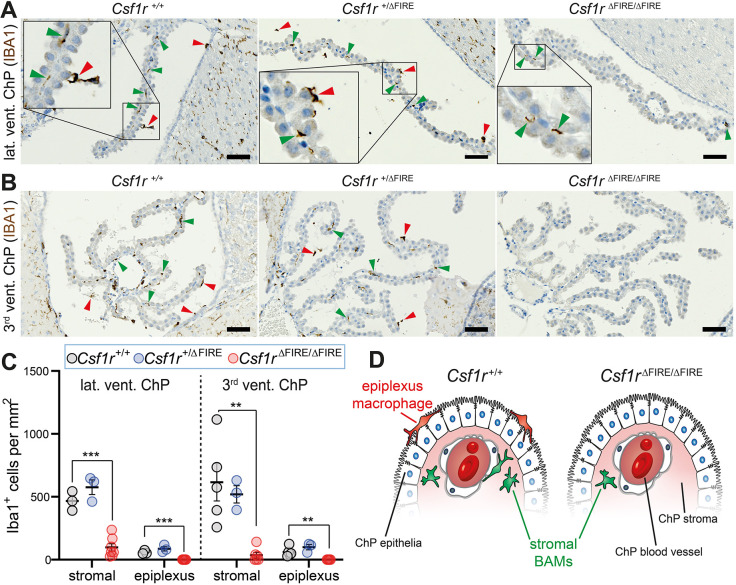
***Csf1r*^ΔFIRE/ΔFIRE^ mice lack intracerebroventricular and ChP macrophages.** (A,B) Adult brains were stained with anti-IBA1 and counterstained with haematoxylin. ChP macrophages were imaged in the (A) lateral (lat.) and (B) third (3rd) ventricles (vent.). Green arrowheads show example stromal ChP BAMs. Red arrowheads show example intraventricular macrophages (epiplexus, supraependymal and free-floating macrophages). (C) Quantification of adult epiplexus and stromal ChP macrophages [*n* (mice)=3-5 *Csf1r^+/+^*; *n*=3 *Csf1r*^+/ΔFIRE^; *n*=6-7 *Csf1r*^ΔFIRE/ΔFIRE^]. Genotypes were compared using one-way ANOVA (apart from third ventricle stromal ChP comparisons, in which Kruskal–Wallis testing was performed). Multiple comparison tests compared *Csf1r*^+/+^ control mice with the other genotypes. **P<0.01, ***P<0.001. Data are mean±s.e.m. (D) Cartoon depicting loss of ChP-associated macrophages in adult *Csf1r*^ΔFIRE/ΔFIRE^ mice. Scale bars: 50 µm.

Within the cerebroventricular system, we show that IBA1^+^ macrophages (epiplexus, supraependymal and free-floating macrophages) were entirely absent in both the lateral and third ventricles of adult *Csf1r*^ΔFIRE/ΔFIRE^ mice, but present in both *Csf1r*^+/+^ and *Csf1r*^+/ΔFIRE^ controls (red arrowheads; Fig. 1A-C). These data demonstrate that, like microglia (Rojo et al., 2019), intracerebroventricular macrophages are dependent on FIRE in adult mice.

In contrast to the self-renewing epiplexus macrophages on the ChP surface, stromal ChP BAMs are gradually replaced over time by bone marrow-derived monocytes (Goldmann et al., 2016; Van Hove et al., 2019). In sections from *Csf1r^+/+^* and *Csf1r*^+/ΔFIRE^ brains, many IBA1^+^ stromal ChP BAMs were present in both the lateral and third ventricles (green arrowheads; Fig. 1A-C). Relative to adult *Csf1r^+/+^* mice, *Csf1r*^ΔFIRE/ΔFIRE^ mice had 20.9%±6.3% (mean±s.e.m.) of the stromal ChP BAMs per mm^2^ in the lateral ventricle and 5.8%±3.7% in the third ventricle (Fig. 1B,C). These results demonstrate that BAMs in this non-parenchymal CNS compartment are only partially dependent on FIRE in adult mice (Fig. 1D).

As the central canal of the spinal cord is continuous with the cerebroventricular system, we next investigated spinal cords of *Csf1r*^ΔFIRE/ΔFIRE^ mice (Fig. S1A). Cervical segments from the spinal cord were isolated and immunostained with anti-IBA1 and fluorescein-labelled *Griffonia simplicifolia* Lectin-I (GSL-I, which labels both blood vessels and the central canal luminal surface). A previous study indicated that macrophages in the central canal were only present during injury (Shechter et al., 2013). Indeed, IBA1^+^ cells were not observed in the central canal of healthy adult *Csf1r*^+/+^, *Csf1r*^+/ΔFIRE^ or *Csf1r*^ΔFIRE/ΔFIRE^ mice (Fig. S1B,C). As with the brain, the spinal cord of *Csf1r*^ΔFIRE/ΔFIRE^ mice maintained some perivascular and meningeal BAMs but no grey or white matter microglia (Fig. S1D,E). Thus, deletion of FIRE has comparable effects on the respective parenchymal and non-parenchymal macrophages of the spinal cord and brain.

Together, these data demonstrate that CNS macrophage loss in adult *Csf1r*^ΔFIRE/ΔFIRE^ mice is not restricted to brain microglia, as intracerebroventricular macrophages and ChP BAMs are also affected. The dependency of both microglia and Kolmer's epiplexus macrophages on FIRE is consistent with recent data suggesting that epiplexus macrophages are more akin to microglia than to BAMs (Van Hove et al., 2019). The same may also be true for other intracerebroventricular macrophages, such as free-floating and supraependymal macrophages, which were also absent in *Csf1r*^ΔFIRE/ΔFIRE^ mice (Fig. 1A,B).

### Developmental arrival of intracerebroventricular macrophages

We next sought to explore whether *Csf1r* mutations influence the initial arrival of primitive microglia and BAMs during development, or whether these macrophages only become CSF1R-dependent after their maturation. To examine this, we first had to understand the normal arrival dynamics of these CNS macrophage populations. The appearance of microglia and cephalic mesenchyme BAMs during embryogenesis is relatively well characterised (Lichanska et al., 1999; Ginhoux et al., 2010; Kierdorf et al., 2013; Utz et al., 2020), so we focused predominately on intracerebroventricular macrophages.

**Fig. 2.**
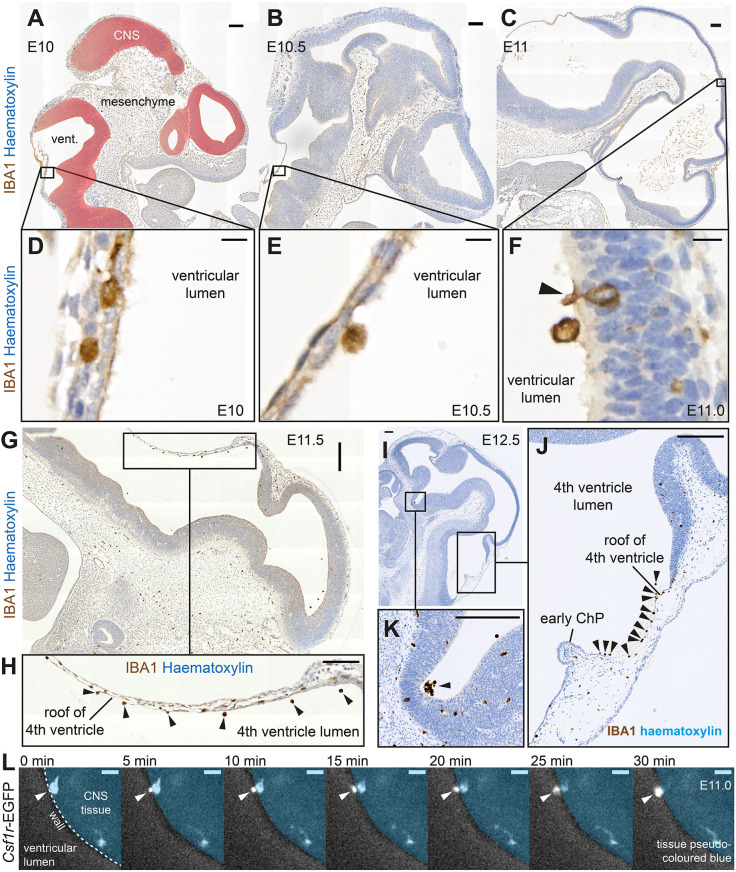
**Arrival dynamics of intracerebroventricular macrophages during normal development.** (A-C) Anti-IBA1-stained sagittal sections from E10, E10.5 and E11 wild-type mouse embryos (representative of *n*=2 C57BL/6JOlaHsd embryos per developmental stage). The CNS (pseudo-coloured red), cerebroventricles (vent.), and cephalic mesenchyme are labelled in A. (D-F) Magnification of boxed regions from A-C. The macrophages in D are situated within the tissue of the fourth ventricle roof. The macrophage in E is attached to the luminal surface of the fourth ventricle roof. The macrophage in F (arrowhead) lies partly within the ventricle and partly within the CNS parenchyma. (G-J) Many intracerebroventricular IBA1^+^ macrophages are present around the fourth ventricle roof at (G) E11.5 and (H-I) E12.5 (arrowheads). (K) Intracerebroventricular macrophages often arrange in clusters (arrowhead) (images in G-J representative of *n*=4 *Csf1r*^+/+^ embryos per developmental stage). (L) *Ex vivo* time-lapse imaging of a transverse 150 µm head slice from an E11 *Csf1r*-EGFP transgenic mouse. Note the macrophage (arrowhead) crossing from parenchyma (pseudo-coloured blue) into ventricular lumen. Scale bars: 100 µm in A-C,H,K-J; 200 µm in G-I; 10 µm in D-F,L.

The most primitive version of the murine cerebroventricular system forms by E9-E9.5 as the neural tube zips shut (Chau et al., 2015; Nikolopoulou et al., 2017). A previous study proposed that macrophages do not arrive in cerebroventricles until E11 in mouse (Sturrock, 1978). To explore intracerebroventricular macrophage arrival in more detail, we examined sections of embryonic mice using the eHistology Atlas (https://www.emouseatlas.org/emap/eHistology). Using this resource, we established that intracerebroventricular cells first appeared in considerable numbers between E10.5 and E11 (Fig. S2A-D). We investigated whether these cells represented macrophages by immunostaining wild-type E10-E11.5 sections with anti-IBA1. IBA1^+^ cells were not observed in cerebroventricles at E10 (Fig. 2A,D), were occasionally present at a low density at E10.5 (Fig. 2B,E), and were observed at increasing densities at E11 (Fig. 2C,F) and E11.5. In E11.5 and E12.5 embryos, many early intracerebroventricular macrophages localised around the thin membranous roof of the fourth ventricle (Fig. 2G-J) and assembled in clusters (Fig. 2K). Examination of sections in the Human Developmental Biology Resource (http://www.hdbr.org/) digital image hub indicated that intracerebroventricular cells arrive at approximately equivalent developmental stages and locations in human embryos (Fig. S3A-D).

We next explored the mode of arrival of murine intracerebroventricular macrophages. As the tissue enveloping the cerebroventricles contains many periventricular macrophages by E11, we hypothesised that macrophages could cross ventricular walls to enter ventricular lumens. In support of this concept, we occasionally identified IBA1^+^ cells in the CNS parenchyma with cellular extensions that stretched across the neuroepithelial lining into ventricular lumens (Fig. 2F). To directly explore this hypothesis, we performed *ex vivo* time-lapse imaging on rostral slices of E11.0 embryos from *MacGreen* mice (*Csf1r-*EGFP transgenic mice; Sasmono et al., 2003). Using this approach, we observed macrophages actively squeezing through the ventricular wall to enter cerebroventricles (Fig. 2L; Movie 1). These data illustrate that macrophages can cross ventricular walls at the stage of their developmental arrival in *ex vivo* slice cultures.

Together, our results show that intracerebroventricular macrophages arrive from E10.5 in mouse, after neural tube closure, and suggest that their route of entry may be (at least in part) by traversing ventricular walls. Notably, these macrophages start arriving before the first ChP begins forming in the fourth ventricle at approximately E12 (Chen et al., 2017), arguing against the ChP stroma being the source of all intracerebroventricular macrophages (a view favoured by Kappers, 1953; Carpenter et al., 1970; Ling, 1983).

### FIRE deletion has disparate effects on the arrival dynamics of primitive microglia and specific BAM populations

**Fig. 3.**
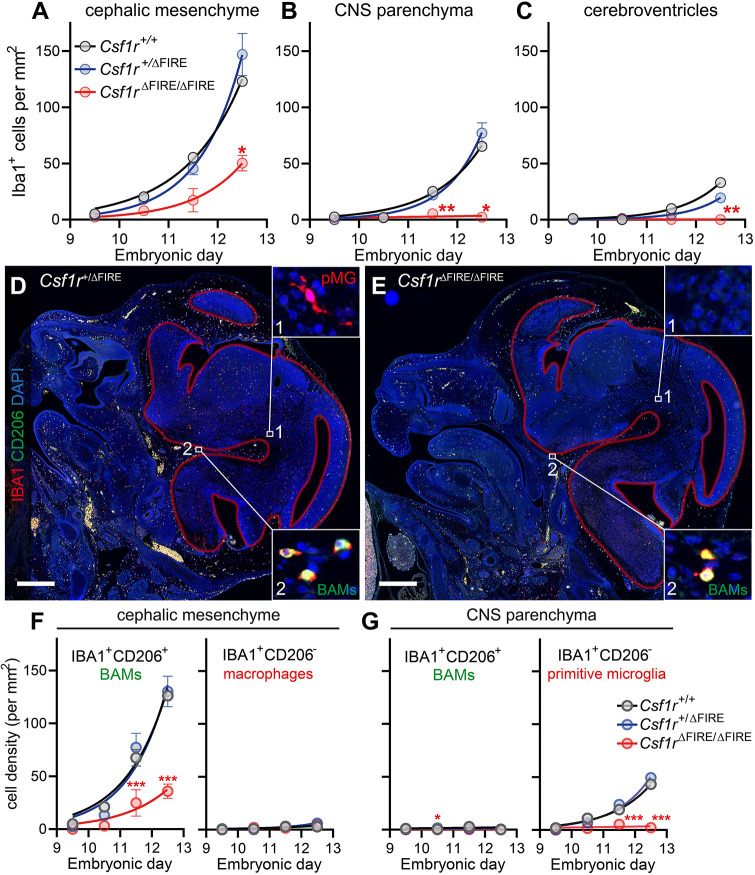
**FIRE deletion differentially impairs microglial and BAM arrival dynamics.** (A-C) Macrophage arrival dynamics in the (A) cephalic mesenchyme, (B) CNS parenchyma and (C) cerebroventricles of *Csf1r^+/+^*, *Csf1r*^+/ΔFIRE^ and *Csf1r*^ΔFIRE/ΔFIRE^ embryos. Number of embryos per genotype (embryos per embryonic day): *Csf1r^+/+^*, 8 (1-4); *Csf1r*^+/ΔFIRE^, 16 (3-5); *Csf1r*^ΔFIRE/ΔFIRE^, 17 (3-5). (D,E) Representative images of E12.5 embryonic heads from (D) *Csf1r*^+/ΔFIRE^ and (E) *Csf1r*^ΔFIRE/ΔFIRE^ mice stained with anti-IBA1 (red), anti-CD206 (green) and DAPI (blue). Auto-fluorescent erythrocytes are also yellow. Red lines depict the borders of the developing CNS tissue. Insets show (1) IBA1^+^CD206^−^ primitive microglia (pMG) and (2) IBA1^+^CD206^+^ BAMs. (F,G) Quantification of IBA1^+^CD206^+^ BAMs and IBA1^+^CD206^−^ primitive microglia in (F) cephalic mesenchyme and (G) CNS parenchyma of *Csf1r^+/+^*, *Csf1r*^+/ΔFIRE^ and *Csf1r*^ΔFIRE/ΔFIRE^ embryos. Number of embryos per genotype (embryos per embryonic day): *Csf1r^+/+^*, 10 (1-5); *Csf1r*^+/ΔFIRE^, 16 (3-4); *Csf1r*^ΔFIRE/ΔFIRE^, 17 (3-5). Mixed-effects analysis with Tukey-corrected multiple comparison post-tests were performed for A-C and F,G. Statistically significant differences compared with the *Csf1r*^+/ΔFIRE^ group are shown (*P<0.05, **P<0.01, ***P<0.001). Data are mean±s.e.m. Scale bars: 500 µm.

Having explored normal embryonic macrophage arrival dynamics, we next examined whether this was affected in *Csf1r*^ΔFIRE/ΔFIRE^ embryos. Consistent with our analyses of C57BL/6JOlaHsd wild-type embryos, cephalic mesenchyme BAMs were the first to arrive in significant numbers in mixed background (C57BL/6J and CBA/Ca) *Csf1r*^+/+^ and *Csf1r*^+/ΔFIRE^ embryos, followed by primitive microglia, and finally intracerebroventricular macrophages (Fig. 3A-C). The arrival dynamics of these macrophage populations (from E9.5 to E12.5) were similar in *Csf1r*^+/+^ and *Csf1r*^+/ΔFIRE^ embryos but clearly impaired in *Csf1r*^ΔFIRE/ΔFIRE^ embryos (Fig. 3A-C). IBA1^+^ cell arrival in the cephalic mesenchyme of *Csf1r*^ΔFIRE/ΔFIRE^ embryos, which includes the meningeal primordium (meninx primitiva), was only partially dampened, whereas the arrival of primitive microglia and intracerebroventricular macrophages was almost entirely prevented (Fig. 3A-C).

**Fig. 4.**
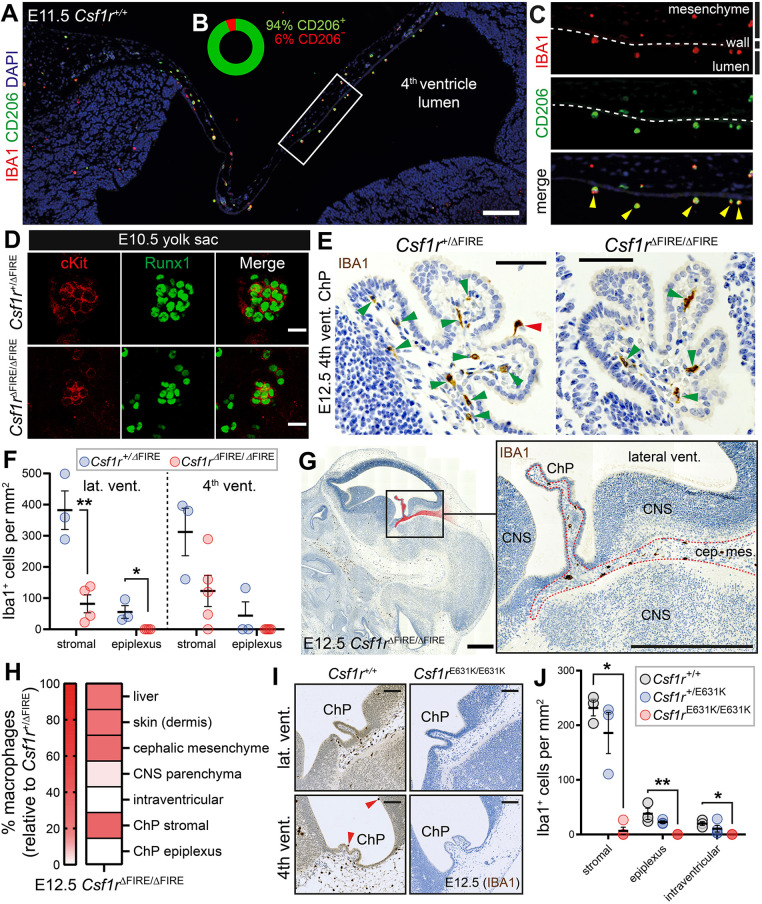
**FIRE deletion differentially impairs the developmental arrival of** **ChP-associated macrophages.** (A) Representative image of an E11.5 embryo showing that most early intracerebroventricular macrophages are IBA1^+^ (red) CD206^+^ (green) cells in the developing ventricular system (*n*=5 *Csf1r*^+/+^ embryos). (B) Percentage of CD206^+^ and CD206^−^ intracerebroventricular macrophages at E11.5 (frequencies based on combined quantification of cells from *n*=5 *Csf1r*^+/+^ and *n*=4 *Csf1r*^+/ΔFIRE^ E11.5 embryos). (C) Magnification of boxed region in A. Yellow arrowheads point to IBA1^+^CD206^+^ intracerebroventricular macrophages. Dashed line indicates the ventricular wall. (D) cKit^+^Runx1^+^ hematopoietic progenitors are generated in the yolk sacs of E10.5 *Csf1r*^ΔFIRE/ΔFIRE^ embryos (representative of *n*=3). (E) Representative images of fourth ventricle ChP of E12.5 *Csf1r*^+/ΔFIRE^ and *Csf1r*^ΔFIRE/ΔFIRE^ embryos stained with anti-IBA1 (brown). Red arrowhead points to epiplexus macrophage, green arrowheads point to stromal macrophages. (F) Quantification of E12.5 ChP macrophage loss [*n* (embryos)=3 *Csf1r*^+/ΔFIRE^; *n=*4-5 *Csf1r*^ΔFIRE/ΔFIRE^). Groups compared using two-tailed *t*-tests or Mann–Whitney tests (*P<0.05, **P<0.01). (G) Presence of IBA1^+^ macrophages in the tissue between cephalic mesenchyme and ChP stroma (pseudo-coloured red and dotted line) of E12.5 *Csf1r*^ΔFIRE/ΔFIRE^ embryos. Boxed area is enlarged on the right. (H) Percentage of macrophage loss in various anatomical regions of E12.5 *Csf1r*^ΔFIRE/ΔFIRE^ embryos (white signifies complete loss and red signifies varying degrees of retention). (I,J) *Csf1r*^E631K/E631K^ embryos are devoid of intracerebroventricular IBA1^+^ macrophages. Data in J from combined quantification of lateral and fourth ventricle ChP macrophages (*n* [embryos]=3 *Csf1r^+/+^*; *n*=3-4 *Csf1r*^+/E631K^; *n=*4 *Csf1r*^E631K/E631K^). *P*-values determined using one-way ANOVA (apart from stromal ChP BAM comparisons, where Kruskal–Wallis testing was performed). Multiple comparison tests compared *Csf1r*^+/+^ control embryos with the other genotypes. (*P<0.05, **P<0.01). Scale bars: 200 µm in A; 20 µm in D; 50 µm in E; 500 µm in G; 100 µm in I.

To verify that the remaining tissue-embedded macrophages in embryonic *Csf1r*^ΔFIRE/ΔFIRE^ heads were BAMs, we co-stained E9.5-E12.5 sections for IBA1 and the BAM-associated marker CD206 (Utz et al., 2020; Fig. 3D,E). The retained macrophages in E9.5-E12.5 *Csf1r*^ΔFIRE/ΔFIRE^ heads were predominantly IBA1^+^CD206^+^ cephalic mesenchyme BAMs (Fig. 3F), whereas IBA1^+^CD206^−^ primitive microglia were largely absent from the CNS parenchyma (Fig. 3G). This specific retention of CD206^+^ BAMs was also observed in the heads of postnatal day 0 *Csf1r*^ΔFIRE/ΔFIRE^ pups (Fig. S4). Notably, the majority of the early intracerebroventricular macrophages in wild-type (*Csf1r*^+/+^) and heterozygous (*Csf1r*^+/ΔFIRE^) embryos abundantly expressed CD206 (Fig. 4A-C), illustrating that these macrophages are not identical to microglia, despite them being similarly reliant on FIRE.

Primitive microglia and BAMs both derive from progenitors generated in the yolk sac (Utz et al., 2020). As macrophages arrived in the cephalic mesenchyme of *Csf1r*^ΔFIRE/ΔFIRE^ embryos (albeit in reduced numbers), we hypothesised that their progenitors must develop in *Csf1r*^ΔFIRE/ΔFIRE^ embryos. Consistent with this hypothesis, clusters of cKit^+^Runx1^+^ hematopoietic progenitors were present in E10.5 *Csf1r*^ΔFIRE/ΔFIRE^ yolk sacs (Fig. 4D). Future studies should investigate the capacity of these cells to proliferate and differentiate in the absence of FIRE.

By E12.5, ChP formation had started in the lateral and fourth ventricles, and stromal and epiplexus ChP macrophages were already present in *Csf1r*^+/+^ and *Csf1r*^+/ΔFIRE^ embryos (Fig. 4E). As with adults, epiplexus macrophages were completely absent from E12.5 *Csf1r*^ΔFIRE/ΔFIRE^ embryos, and the stromal ChP BAMs were also considerably reduced (Fig. 4E,F). These results were substantiated using two other macrophage markers, F4/80 and CD206 (Fig. S5A-I), which are more abundantly expressed by early BAM-lineage cells than microglia-lineage cells (Utz et al., 2020).

The stroma of the ChP is continuous with streams of cephalic mesenchyme, which subsequently develops into meningeal tissue (Fig. 4G). Accordingly, the ChP stroma is often considered as invaginated meningeal tissue (Kappers, 1953; Dziegielewska et al., 2001). Notably, the partial loss of stromal ChP BAMs in E12.5 *Csf1r*^ΔFIRE/ΔFIRE^ embryos corresponded closely to the partial loss of cephalic mesenchyme/meningeal primordium BAMs (Fig. 4H). Macrophages in non-CNS peripheral tissues, such as liver and dermis, were also reduced to a similar extent (Fig. 4H; Fig. S6). In contrast, the complete loss of intracerebroventricular macrophages at E12.5 corresponded more closely to the absence of parenchymal CNS macrophages (2.9±0.5% relative to *Csf1r*^+/ΔFIRE^, with most of the remaining macrophages appearing to be perivascular).

To confirm that stromal ChP BAMs and intracerebroventricular macrophages are dependent upon CSF1R signalling, we examined mouse embryos harbouring a kinase-dead *Csf1r* mutation, E631K (Pridans et al., 2013). Heterozygous *Csf1r*^E631K/+^ embryos model a dominant human *CSF1R* mutation associated with ALSP (Rademakers et al., 2011). Homozygous *Csf1r*^E631K/E631K^ mice closely resemble *Csf1r* knockout mice (Erblich et al., 2011), with significant embryonic macrophage loss, severe postnatal growth retardation, and pre-weaning mortality (D.A.H. and C.P., unpublished). At E12.5, we show that intracerebroventricular macrophages were completely absent in *Csf1r*^E631K/E631K^ mice (Fig. 4I,J). E12.5 *Csf1r*^E631K/E631K^ embryos also exhibited a more substantial loss of stromal ChP BAMs compared with *Csf1r*^ΔFIRE/ΔFIRE^ embryos (Fig. 4I,J).

In summary, we show that CNS macrophage populations differ in their dependency on the FIRE enhancer. The arrival and maintenance of microglia and intracerebroventricular macrophages are reliant on FIRE, whereas cephalic mesenchyme and stromal ChP BAMs are only partially dependent. Our results are consistent with a recent study showing that primitive BAMs and microglia segregate from yolk sac progenitors into two differentially regulated, transcriptionally distinct macrophage populations early in their development (Utz et al., 2020). Our data also agree with recent evidence from both rodent and human studies showing that certain populations of cerebrospinal fluid-exposed macrophages share properties with microglia (Farhadian et al., 2018; Van Hove et al., 2019; Schafflick et al., 2020; Esaulova et al., 2020). Even when considering these similarities, we suggest that the macrophage populations of the cerebrospinal fluid system (or CSF macrophages, collectively) should be considered as a third category of CNS macrophages (alongside microglia and BAMs), rather than as a microglial subset.

## MATERIALS AND METHODS

### Animals

Mice of both sexes were maintained and bred under specific pathogen-free conditions in accordance with the institutional guidelines and regulations as set by The University of Edinburgh. Mice were culled by qualified staff of a UK Home Office-licensed animal house following guidelines set under Schedule 1 of the UK Animals (Scientific Procedures) Act 1986. Sample sizes are provided in respective figure legends. The spinal cord analyses used 7- to 12-week-old mice and 4- to 39-week-old mice were used for the ChP analyses.

Wild-type tissues in Fig. 2A-F were obtained from inbred C57BL/6JOlaHsd mice purchased from Envigo. The published transgenic strains used in this study were *Csf1r*-EGFP (Sasmono et al., 2003) and *Csf1r*^ΔFIRE^ (Rojo et al., 2019). The generation of *Csf1r*^E631K^ mice will be described in a separate publication (D.A.H. and C.P., unpublished). *Csf1r*-EGFP males (>10 backcrosses onto C57BL/6JOlaHsd background) were used for timed matings at 2-12 months old. *Csf1r*-EGFP embryos were generated by timed matings of C57BL/6JOlaHsd wild-type females with *Csf1r*-EGFP males. Genotyping was performed by EGFP fluorescence screening. The *Csf1r*^ΔFIRE^ mice used in this study were on a mixed C57BL/6JCrl and CBA/Ca genetic background (Rojo et al., 2019).

For embryonic experiments, the morning of vaginal plug discovery was considered as E0.5 and staging was verified by somite pair (SP) counting of embryos up to E11.5 (E9.5=21-29 SP; E10=30-34 SP; E10.5=35-39 SP; E11=40-44 SP; E11.5=45-47 SP). For embryos >E11.5, staging accuracy was validated based on assessment of anatomical landmarks (Kaufman, 1992), as SP counting is more difficult and less meaningful after E11.5 (Wong et al., 2015).

### Immunostaining

#### Immunohistochemistry

Tissues were isolated, immersion fixed in 4% buffered formaldehyde for 1-2 days, dehydrated and embedded in paraffin blocks. For immunostaining, 6 µm tissue sections were de-waxed using xylene (2×5 min), rehydrated through an ethanol:dH_2_O series (100% and 50% for 2 min each) and rinsed in tap water for 5 min. For IBA1 staining, antigen retrieval was performed using Vector antigen unmasking solution (H-3300; Vector Laboratories; according to manufacturer's instructions). Sections were then rinsed in phosphate-buffered saline (PBS; 2×2 min) before endogenous peroxidases were blocked using 3% hydrogen peroxide:dH_2_O for 10 min. Slides were rinsed with PBS and non-specific protein binding was blocked using 2.5% horse serum (S-2012, Vector Laboratories) for 20 min at room temperature (RT). Sections were subsequently incubated for 40 min in anti-IBA1 polyclonal rabbit antibody (1:500; Wako, 019-19741) in 2.5% horse serum, rinsed with PBS (3×5 min) and then incubated for 35 min with the ImmPRESS^®^ peroxidase (HRP) polymer anti-rabbit IgG reagent (produced in horse; MP-7801, Vector Laboratories). Sections were rinsed (PBS, 2×5 min) and incubated with an ImmPACT^®^ DAB HRP substrate for 5 min (SK-4105; Vector Laboratories; as per manufacturer's instructions). Finally, sections were rinsed in tap water, counterstained with haematoxylin, dehydrated and cover-slipped. The following modifications were made for F4/80 staining (1:500; AbD Serotec, MCA497G): antigen retrieval was performed using proteinase K [20 µg/ml in TE buffer, (pH 8)] at 37°C for 5 min; 2.5% normal goat serum (S-1012, Vector Laboratories) was used for blocking steps; the ImmPRESS^®^ HRP goat anti-rat IgG kit (MP-7444; Vector Laboratories) was used for primary antibody detection. For co-immunofluorescence staining for CD206 and IBA1, the following modifications were made: antigen retrieval was performed using antigen retrieval buffer (HDS05-100; TCS Biosciences, according to manufacturer's instructions); 10% goat serum and 0.1% Triton X-100 in PBS was used for blocking steps; primary antibody [anti-CD206 (1:200; R&D Systems, AF2535) and anti-IBA1 (1:500; Wako, 019-19741)] and secondary antibody [Alexa Fluor 488 (A-11055, 1:200) and 568 (A10042, 1:200)] incubations were performed overnight at 4°C; sections were counterstained with DAPI for 3 min.

#### Spinal cord immunofluorescence staining

All animals (7-12 weeks of age) were terminally anaesthetised via a lethal dose of Euthatal (at 0.3 ml/100 g bodyweight by intraperitoneal injection) and rapidly perfused with cold PBS as a prewash followed by cold 4% paraformaldehyde in PBS. The cerebral (C)8 vertebra of the spinal cord was identified following detection of the large dorsal process and C4-C7 vertebrae. Vertebrae were removed and post-fixed in 4% paraformaldehyde overnight before being transferred to a 25% sucrose solution in PBS for cryoprotection. Spinal segments were frozen in Tissue-Tek OCT compound (Leica) and 16 μm transverse sections were cut on a cryostat and placed on SuperFrost Plus glass slides (VWR International) and stored at −80°C. Sections were blocked for 1 h using 3% normal goat serum in PBS-triton (0.2% Triton X-100 in PBS). Sections were then incubated with anti-IBA1 and fluorescein-labelled GSL-I (1:150, FL-1101, Vector Laboratories) in PBS-Triton with 1% normal goat serum overnight at RT. Slides were then washed several times in PBS and incubated with the goat anti-rabbit Alexa Fluor-555 secondary antibody (1:1000; Invitrogen, A27039) to identify anti-IBA1. Secondary antibodies were added for 2 h in PBS containing 1% normal goat serum and bis-benzamide to identify cell nuclei (1:5000, Sigma-Aldrich). Slides were rinsed in PBS and subsequently rinsed for 2×10 min in tris-non-buffered saline to remove any remaining salts, before being cover-slipped using Fluorsave Reagent (Calbiochem).

#### Yolk sac whole-mount staining

Yolk sacs were separated from embryos and fixed with 4% buffered formaldehyde for 1 h. Samples were rinsed in PBS (30 min) and dehydrated in a methanol:dH_2_O series (25%, 50%, 75% and 100% for 15 min each). Samples were then stored at −20°C or directly processed. Rehydration of samples was performed using a methanol:dH_2_O series (100%, 75%, 50% and 25% for 15 min each). The tissue was rinsed in PBS and blocked with PBS with 5% donkey serum (Sigma-Aldrich, D9663) for 2 h at RT. Yolk sacs were incubated with anti-c-Kit (1:200, clone 2B8, BD Biosciences) and anti-Runx1 (1:200, ab92336; Abcam) overnight at 4°C, rinsed in PBS (4×30 min), then incubated with Alexa Fluor secondary antibodies (A-21206, 1:200; A-11077, 1:200; Invitrogen) overnight at 4°C). Yolk sacs were rinsed with PBS, placed on slides and covered in mounting medium (Vectashield; Vector Laboratories, H1000), then cover-slipped.

### Microscopy and image analysis

Embryonic and adult brain sections were imaged using a Zeiss AxioScan.Z1 microscope (20× objective with automated tissue detection and automated stitching of images by Zen software during the image acquisition). Spinal cord sections and whole-mount yolk sacs were imaged using a Zeiss LSMZ10 confocal microscope. Macrophage density quantifications (IBA1, CD206 and F4/80 cells per mm^2^) were calculated by dividing macrophage number by the area of the tissue region. Macrophage numbers were manually counted using plugin→analyse→cell counter in ImageJ. Tissue areas were calculated using the spline contour tool in Zen 2.6 (blue edition). An Andor spinning disk confocal microscope (Andor Revolution XDi) was used for *ex vivo* time-lapse imaging.

### *Ex vivo* time-lapse imaging

Rostral parts of transgenic *Csf1r*-EGFP embryos were cut transversally (150 µm sections) with an automated tissue-chopper (McIlwain tissue chopper). Suitable sections were transferred into a chamber ring support (Attofluor cell chamber, Invitrogen) that was sealed with a glass cover slip (Menzel Glaser, VWR International). Sections were then embedded in 1.5% low melting agarose (Sigma-Aldrich). Agarose-embedded sections were incubated with MyeloCult M5300 medium (Stemcell Technologies) supplemented with hydrocortisone succinate 10^−6^ M (Sigma-Aldrich) and 20 ng/ml murine IL3 (Peprotech). Sections were imaged overnight using an Andor spinning disk (20× objective; 5 min intervals between each *z*-stack) at a temperature of 37°C in a 5% CO_2_ environment. Lateral drift correction was performed using ImageJ. Movie 1 was prepared using Adobe Premiere Pro (2019).

### Databases

The eHistology atlas (https://www.emouseatlas.org/emap/eHistology) was used to assess the arrival timing of intracerebroventricular cells in mouse embryonic development. Cells per section were manually quantified from various sagittal and transverse sections at each relevant embryonic age. To assess the arrival timing of intracerebroventricular cells in human embryonic development, we were granted access to images of transverse sections of human embryos from the human developmental biology resource (http://www.hdbr.org/).

### Statistical analyses

Details of statistical tests used to compare groups can be found in respective figure legends. *P*<0.05 was considered as statistically significant (*P<0.05, **P<0.01, ***P<0.001). Power calculations were not performed to determine sample sizes. Data collection and analyses were not performed blind to the experimental conditions. All graphs show mean±s.e.m. No outliers were removed. Data distributions were subjectively assessed, were tested for normality using Shapiro-Wilk testing, and non-parametric statistical tests were used to compare groups where deemed appropriate (as normality tests lack power to detect non-Gaussian distributions with small data sets, *P*-values obtained from non-parametric tests may be too high, and from parametric tests may be too low and/or inaccurate). All graphs were generated using GraphPad Prism 8.

## Supplementary Material

Supplementary information
