# Supplementary material for: CNS macrophages differentially rely on an intronic Csf1r enhancer for their development
Source: Development. 2020 Dec 16;147(23):dev194449. doi: 10.1242/dev.194449 (PMC7758622; doi:10.1242/dev.194449)
Supplement: Supplementary information [file develop-147-194449-s1.pdf]

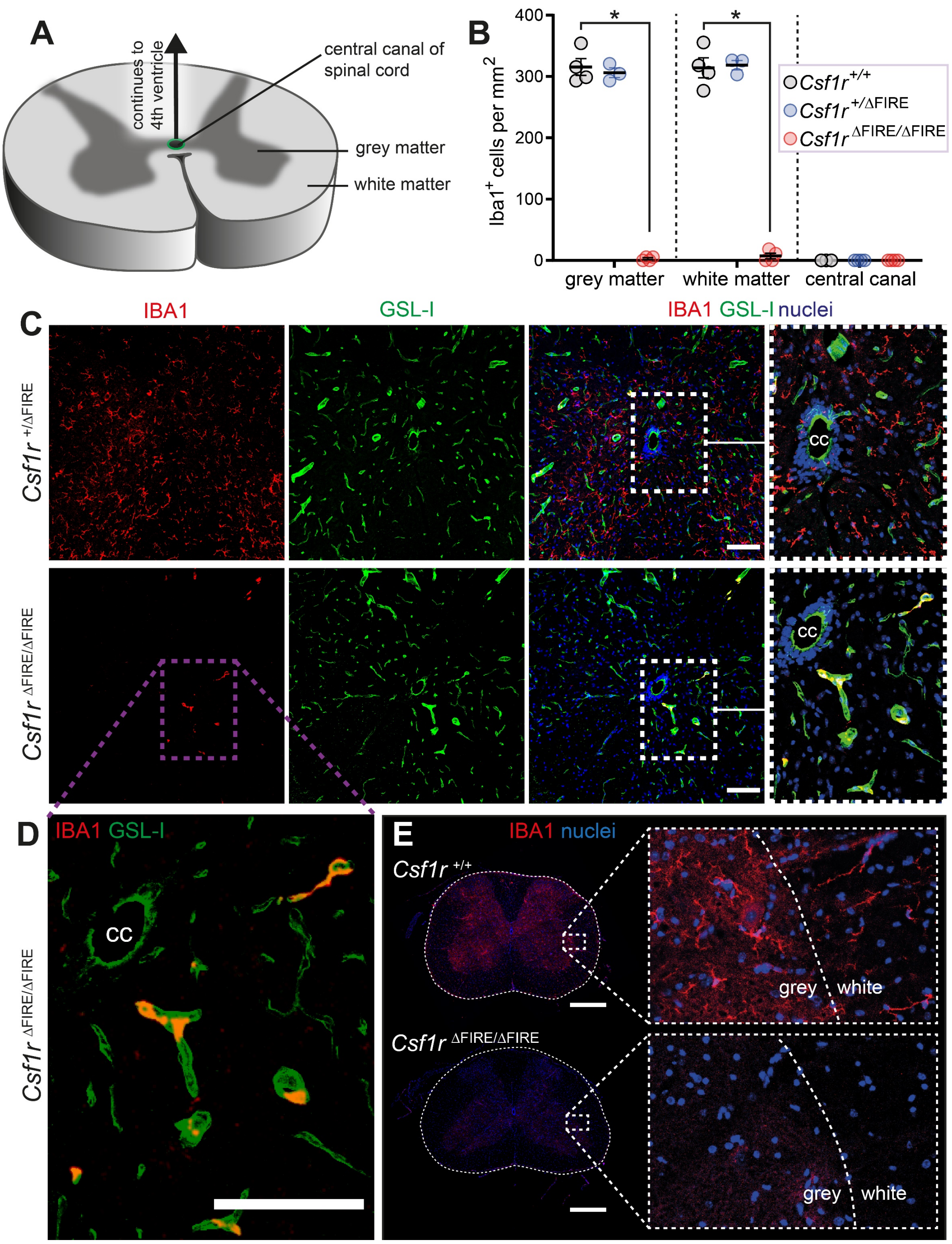

Fig. S1. Spinal cord macrophage loss in *Csf1r*$^{ΔFIRE/ΔFIRE}$ mice. (**A**) Cartoon depicting regions of interest in cross section of spinal cord. (**B**) Quantification of grey matter, white matter, and central canal macrophages in the adult mouse cervical spinal cord (n [mice] = 4 *Csf1r*$^{+/+}$; n = 3 *Csf1r*$^{+/ΔFIRE}$; n = 4 *Csf1r*$^{ΔFIRE/ΔFIRE}$). For the central canal analysis, 9-12 sections were quantified per mouse (16 µm per section). Genotypes were compared using Kruskal-Wallis testing and multiple comparison tests (to compare *Csf1r*$^{+/+}$ controls with the other genotypes). (**C**) Representative images showing grey matter parenchyma and the central canal of the cervical spinal cord. Sections were stained with anti-IBA1 and fluorescein-labelled GSL-I. cc, central canal. (**D**) Some perivascular BAMs are present in spinal cords of *Csf1r*$^{ΔFIRE/ΔFIRE}$ mice. (**E**) Representative sections showing spinal cord microglia in *Csf1r*$^{+/+}$ mice and absence of these cells in *Csf1r*$^{ΔFIRE/ΔFIRE}$ mice. Scale bars: **C-D**, 100 µm; **E**, 500 µm.

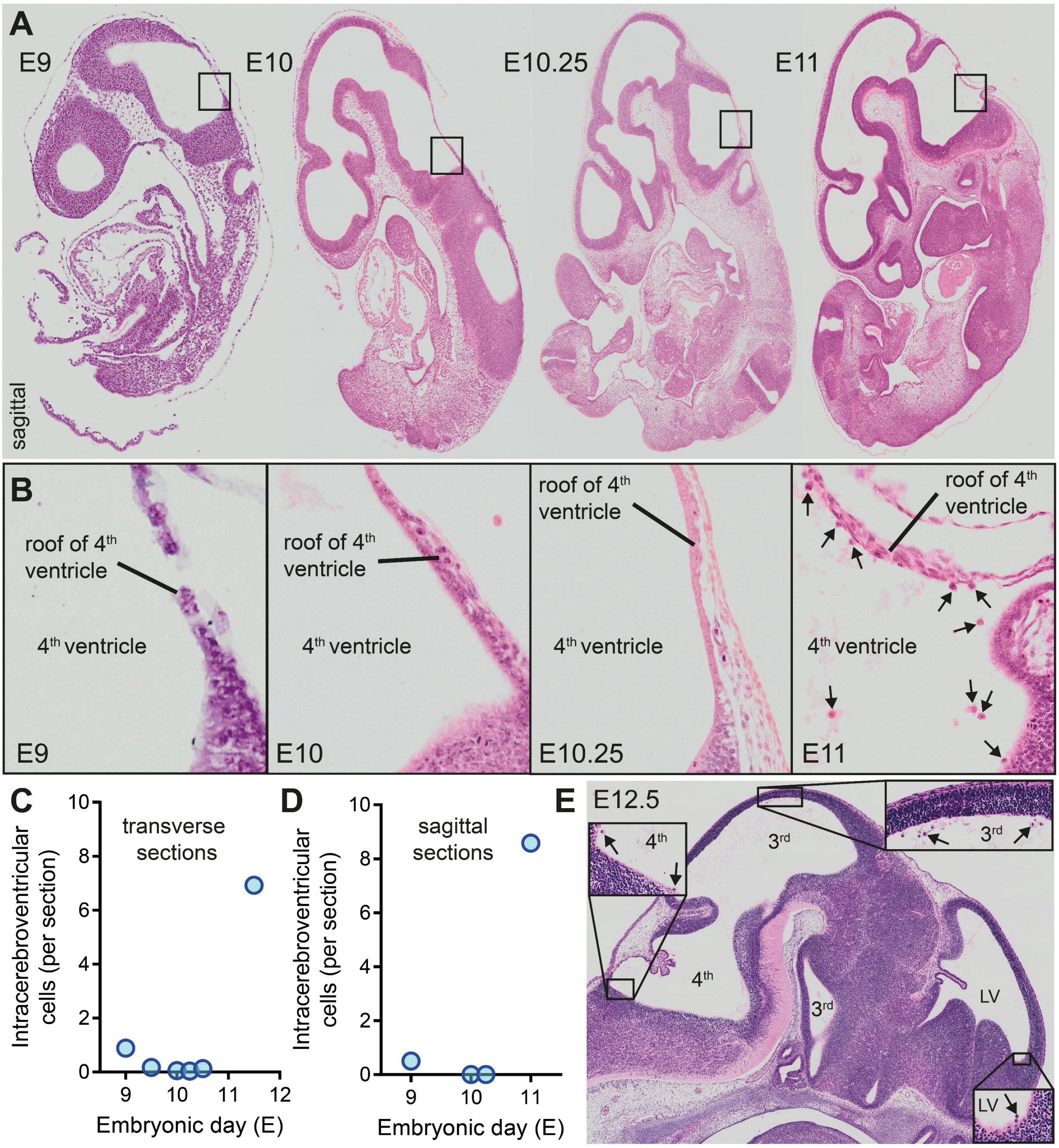

Fig. S2. Intracerebroventricular cell arrival in mouse embryos. (**A**) Images from the eHistology atlas (https://www.emouseatlas.org/emap/eHistology) of E9 to E11 mouse embryos. (**B**) Magnified versions of the boxed regions shown in **A**. Black arrows indicate intracerebroventricular cells. (**C-D**) Quantification of intracerebroventricular cells in (**C**) transverse and (**D**) sagittal sections of mouse embryos in the eHistology atlas (n = 3-26 sections analysed per embryo, 1 embryo per developmental stage). (**E**) Examples of intracerebroventricular cells (black arrows) throughout the cerebroventricular system of an E12.5 mouse embryo.

Development • Supplementary information

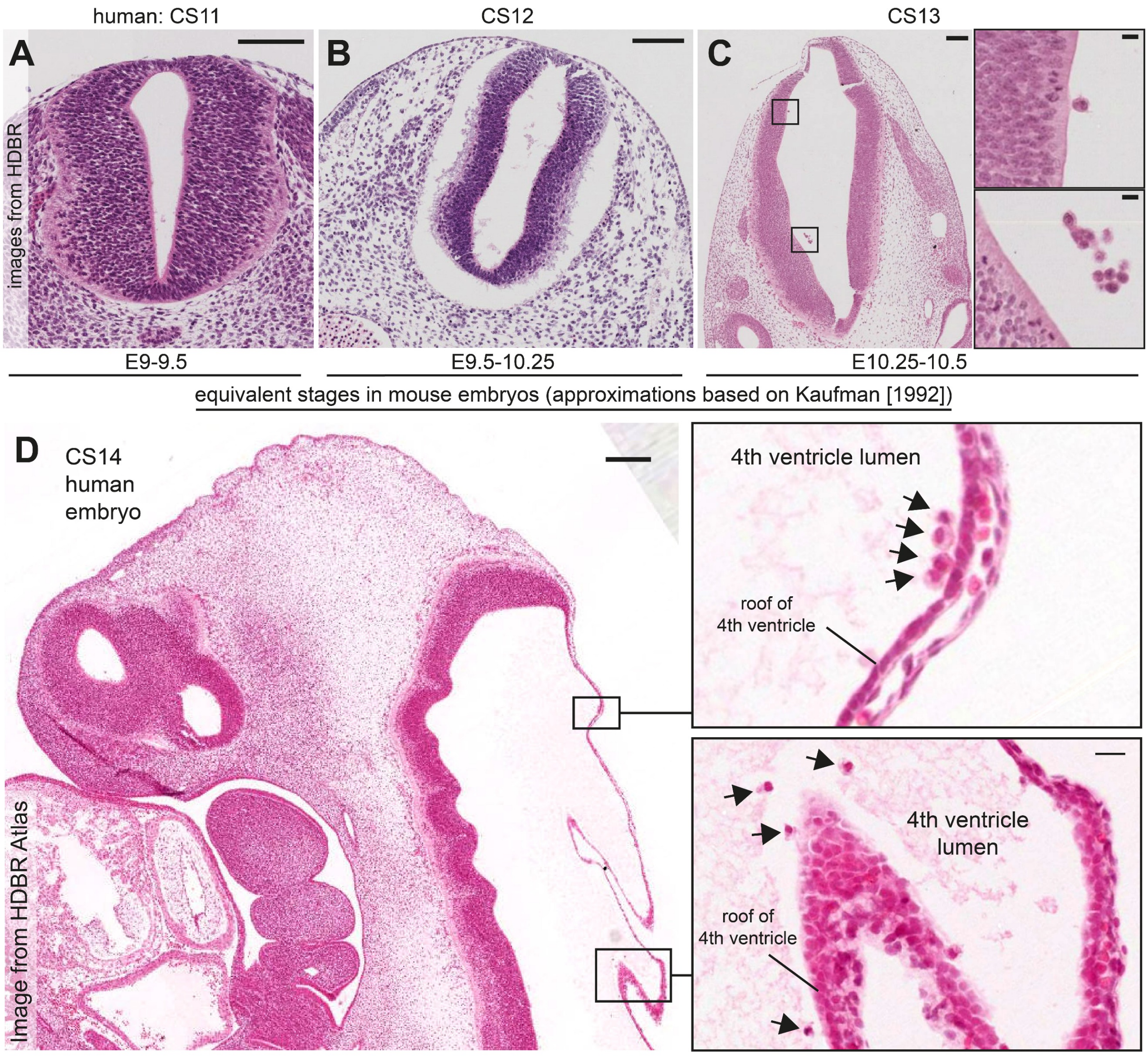

Fig. S3. Intracerebroventricular cell arrival in human embryos. (**A-C**) Representative transverse sections of Carnegie stage (CS)11-13 human embryos from the human developmental biology resource (http://www.hdbr.org/). Consistent with Monier et al. (2007), intracerebroventricular cells were frequently detected in human embryos from CS13 (equivalent to ~E10.25-10.5 in mouse) but not prior to this stage. CS13 intracerebroventricular cells were often observed in clusters (magnified region in **C**). (**D**) CS14 human embryo. Magnified regions show many intracerebroventricular cells adjacent to the fourth ventricle roof. Scale bars: **A-C**, 100 μm; **D**, full image (left) = 200 μm and magnified boxes (right) = 20 μm.

Development • Supplementary information

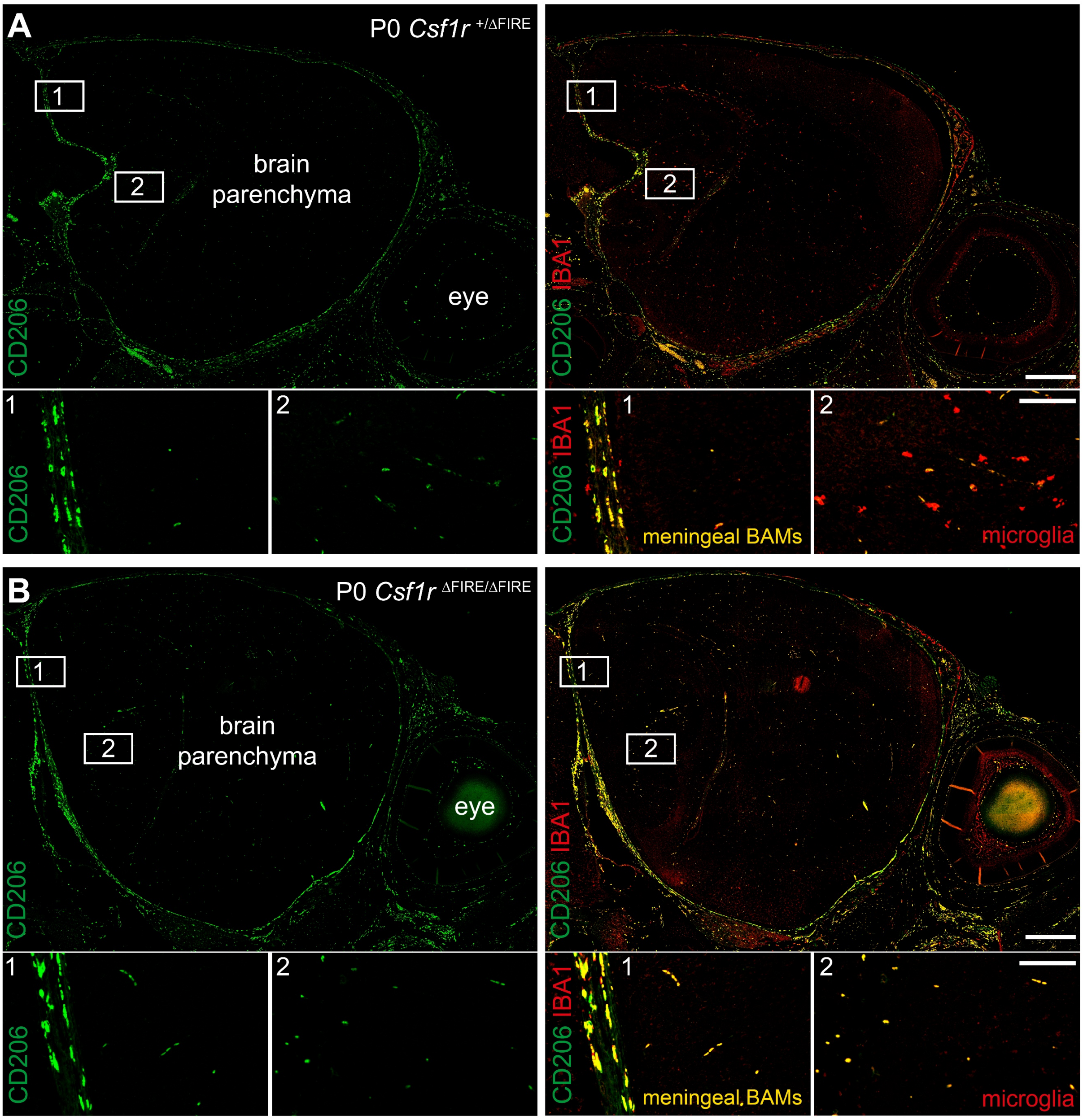

Fig S4. Specific retention of IBA1⁺CD206⁺ BAMs in the CNS of postnatal day 0 *Csf1r*$^{\Delta FIRE/\Delta FIRE}$ pups. (**A-B**) Postnatal day (P)0 *Csf1r*$^{\Delta FIRE/\Delta FIRE}$ pups retain IBA1^{+}CD206^{+} meningeal BAMs, which surround the developing brain parenchyma. IBA1^{+}CD206^{-} parenchymal microglia are not present in *Csf1r*$^{\Delta FIRE/\Delta FIRE}$ pups. A1 and B1 show magnified versions of the white boxes labelled with (**1**). A2 and B2 show magnified versions of the white boxes labelled with (**2**). Note that auto-fluorescing erythrocytes are also labelled in the red and green channels. Representative images from n = 5 *Csf1r*$^{+/\Delta FIRE}$ pups and n = 3 *Csf1r*$^{\Delta FIRE/\Delta FIRE}$ pups. Scale bars: overview images, 500 µm; magnified regions, 100 µm.

Development • Supplementary information

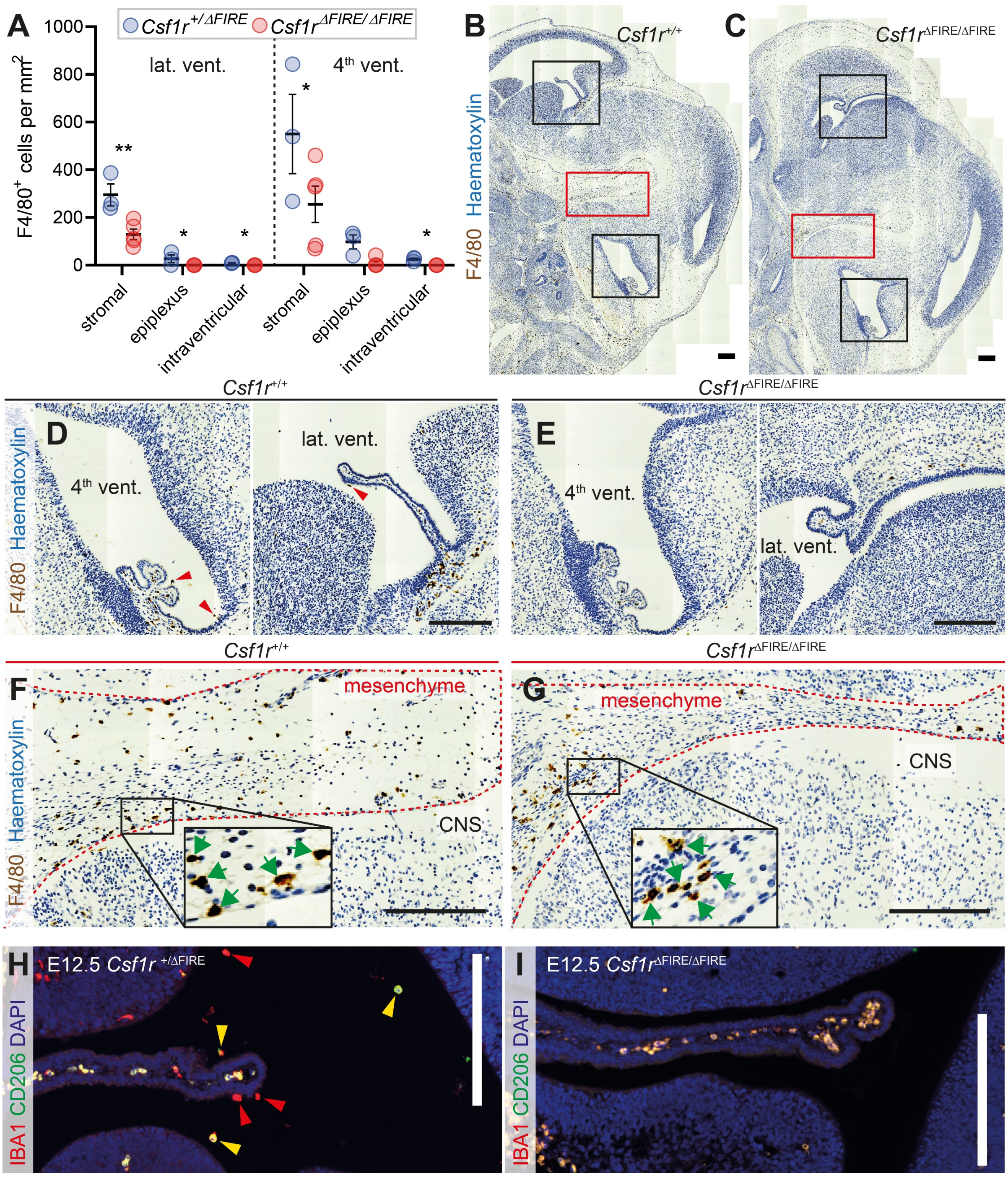

Fig. S5. F4/80 and CD206 staining of macrophages in E12.5 *Csf1r*$^{+/+}$, *Csf1r*$^{+/\Delta FIRE}$, and *Csf1r*$^{\Delta FIRE/\Delta FIRE}$ embryos. (**A**) Quantification of F4/80^{+} macrophage densities in different CNS compartments (n [embryos] = 3-4 *Csf1r*$^{+/\Delta FIRE}$; n = 4-5 *Csf1r*$^{\Delta FIRE/\Delta FIRE}$). Means ± SEM. Groups compared using two-tailed t-tests or Mann-Whitney tests (*p<0.05, **p<0.01). (**B-C**) Representative images of E12.5 *Csf1r*$^{+/+}$ and *Csf1r*$^{\Delta FIRE/\Delta FIRE}$ embryonic heads stained for F4/80. (**D-E**) Anti-F4/80 stained ChP of the lateral (lat.) and fourth (4th) ventricles (these images show magnified versions of the black boxed regions in **B-C**). (**F-G**) F4/80^{+} macrophages (brown) in the cephalic mesenchyme and developing CNS tissue (these images show magnified versions of the red boxed regions in **B-C**) Inset boxes in **F** and **G** show magnified images of cephalic mesenchyme F4/80^{+} BAMs (green arrows). (**H-I**) Representative images of anti-Iba1 (red) and anti-CD206 (green) double-stained ChP macrophages of the lateral ventricle in *Csf1r*$^{+/\Delta FIRE}$ and *Csf1r*$^{\Delta FIRE/\Delta FIRE}$ embryos. Red arrowheads = IBA1^{+}CD206^{-} macrophages; yellow arrowheads = IBA1^{+}CD206^{+} macrophages. Note that erythrocytes are also fluorescing yellow in the ChP stroma. Scale bars = 200 µm.

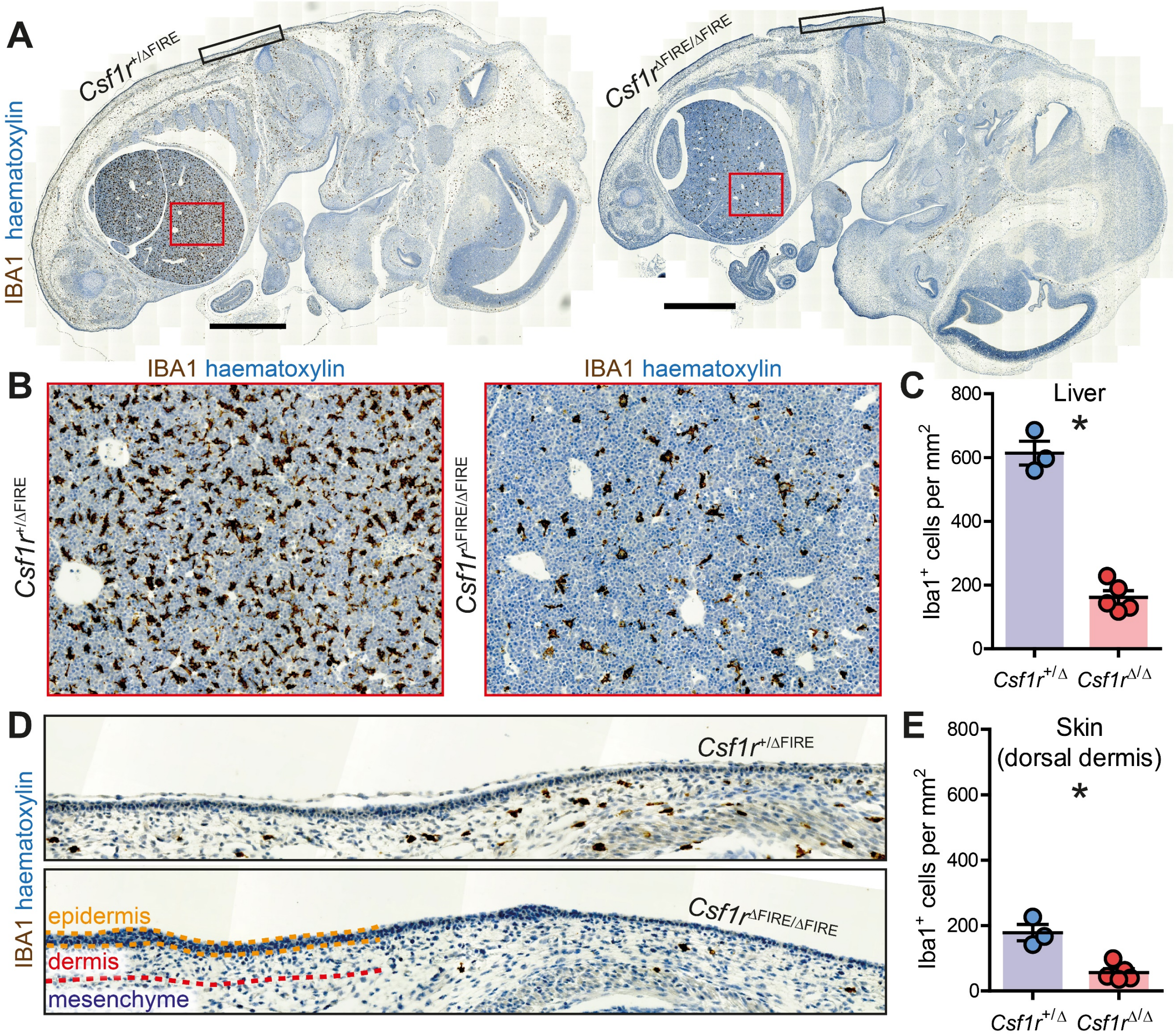

Fig. S6. Loss of peripheral macrophage populations (liver and dorsal trunk dermis) in E12.5 *Csf1r*$^{\Delta FIRE/\Delta FIRE}$ mice. (**A**) Stitched representative overview images of IBA1-stained entire E12.5 *Csf1r*$^{+/\Delta FIRE}$ and *Csf1r*$^{\Delta FIRE/\Delta FIRE}$ embryos (n [embryos] = 3 *Csf1r*$^{+/\Delta FIRE}$; n = 5 *Csf1r*$^{\Delta FIRE/\Delta FIRE}$). Magnified images of the boxed regions (red boxes, liver; black boxes, dermis) are shown in **B** and **D**. (**B**) Representative images showing IBA1^{+} liver macrophages in E12.5 *Csf1r*$^{+/\Delta FIRE}$ versus *Csf1r*$^{\Delta FIRE/\Delta FIRE}$ embryos. (**C**) Quantification of E12.5 liver macrophages in *Csf1r*$^{+/\Delta FIRE}$ and *Csf1r*$^{\Delta FIRE/\Delta FIRE}$ embryos. (**D**) Representative images of IBA1^{+} skin macrophages in E12.5 *Csf1r*$^{+/\Delta FIRE}$ versus *Csf1r*$^{\Delta FIRE/\Delta FIRE}$ embryos. (**E**) Quantification of E12.5 dorsal dermal macrophages in *Csf1r*$^{+/\Delta FIRE}$ and *Csf1r*$^{\Delta FIRE/\Delta FIRE}$ embryos. Two-tailed Mann-Whitney tests were used to compare groups in **c** and **e** (*$p<0.05$). Scale bars = 1 mm.

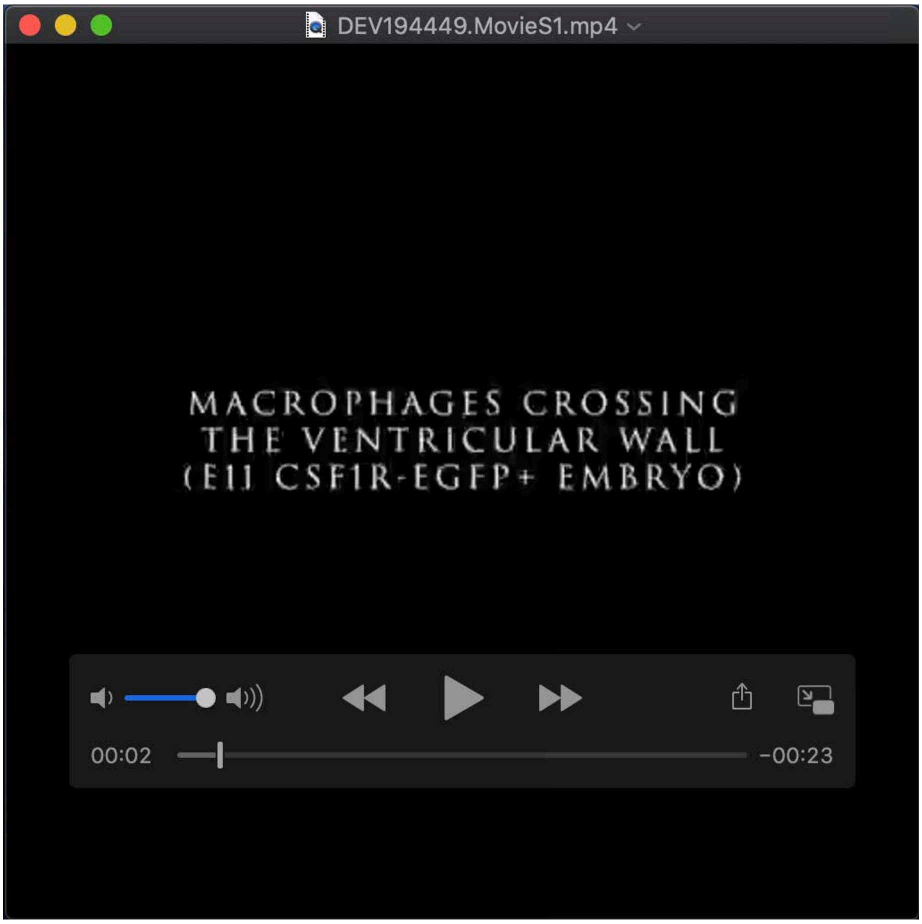

Movie 1. *Csf1r*-EGFP macrophages crossing the ventricular wall and entering the cerebroventricular lumen (2 cell crossing events were observed from live-imaging of 7 embryonic slice cultures).
